# Case report: a rare case of Hunter syndrome (type II mucopolysaccharidosis) in a girl

**DOI:** 10.1186/s12881-019-0807-x

**Published:** 2019-05-02

**Authors:** A. N. Semyachkina, E. Y. Voskoboeva, E. Y. Zakharova, E. A. Nikolaeva, I. V. Kanivets, A. D. Kolotii, G. V. Baydakova, M. N. Kharabadze, R. G. Kuramagomedova, N. V. Melnikova

**Affiliations:** 1 Department of Clinical Genetics, Research and Clinical Institute of Pediatrics named after Yuri Veltischev of the Pirogov Russian National Research Medical University of the Ministry of Health of the Russian Federation, 2 Taldomskaya St, Moscow, 125412 Russia; 2 Research Centre for Medical Genetics RAN, 1 Moskvorechie St, Moscow, 115522 Russia; 3 Genomed, Moscow, 8/5 Podolskoye Shosse, Moscow, Russia; 4 Kuban Medical and Genetics Center, 167, Pervogo Maya St, Krasnodar, Russia

**Keywords:** Мucopolysaccharidosis, Hunter syndrome, Turner syndrome, Iduronate 2-sulfatase, *IDS* gene

## Abstract

**Background:**

Hunter syndrome (mucopolysaccharidosis type II) is a recessive X-linked disorder due to mutations in the iduronate 2-sulfatase (*IDS)* gene. The *IDS* gene encodes a lysosomal enzyme, iduronate 2-sulfatase. The disease occurs almost exclusively in males. However, in the literature, 12 cases of the disease in females are known due to structural anomalies, a non-random chromosome X inactivation or chromosome X monosomy. The purpose of this article is to demonstrate a rare case of Hunter syndrome in a girl caused by a mutation in the *IDS* gene inherited from the mother and the presence of chromosome X of paternal origin, partially deleted in the long arm region - 46,X,del(X)(q22.1).

**Case presentation:**

Girl M., 4 years old, entered the hospital with growth retardation, pain in the lower limbs, and joint stiffness, noted from the age of 18 months. After the karyotype analysis, which revealed a partial deletion of the long arm of chromosome X - 46, X, del (X) (q 22.1), Turner syndrome was diagnosed. However, due to the hurler-like facial phenotype, Hurler syndrome or type I mucopolysaccharidosis (MPS) was suspected. The study of lysosomal enzymes showed normal alpha-L-iduronidase activity and a sharp decrease in the activity of iduronate sulfatase in the blood: 0.001 μM/l/h, at a rate of 2.5–50 μM/l/h. Molecular genetic analysis revealed a hemizygous deletion in the *IDS* gene, which was not registered in the international Human Gene Mutation Database (HGMD) professional. This deletion was not detected in the girl’s father, but was detected in her mother in the heterozygous state.

**Conclusions:**

Thus, the girl confirmed comorbidity - Turner syndrome with a partial deletion of the long arm of chromosome X of paternal origin, affecting the Xq28 region (localization of the *IDS* gene), and Hunter syndrome due to a deletion of the *IDS* gene inherited from the mother. The structural defect of chromosome X in the girl confirmed the hemizygous state due to the mutation in the *IDS* gene, which has led to the formation of the clinical phenotype of Hunter syndrome.

## Background

Among the hereditary progressive diseases of childhood, accompanied by the defeat of the leading organs and body systems, mucopolysaccharidoses occupy one of the priority places.

The great phenotypic similarity of this group of diseases, as a rule, causes significant difficulties in identifying types of the disease, which is usually impossible without the use of a complex of modern clinical, biochemical and molecular genetic studies.

Among this group of diseases, Hunter syndrome or mucopolysaccharidosis type II (MPS II, Online Mendelian Inheritance in Man (OMIM) 309,900) is the most known and frequent nosological form. In the history of the study of Hunter syndrome, three important stages should be identified: 100 years since the date of the first description of the disease; 10 years since the beginning of enzyme replacement therapy and less than a year (the end of 2017) since the first in the history of medicine editing of the genome of a 49 year old patient with this monogenic pathology [1].

Hunter syndrome refers to rare diseases, its frequency, according to different researchers, ranges from 0.3 to 0.71 per 100,000 live births [2].

Hunter syndrome differs from other types of mucopolysaccharidosis by recessive type of inheritance linked to chromosome X. On this basis, the vast majority of patients with Hunter syndrome are males. However, there were at least 18 cases of the disease described in girls. Most of them are associated with the *IDS* gene mutation and inactivation disorders of chromosome X, chromosomal translocations, or chromosome X monosomy due to a partial de novo deletion of the long arm of chromosome X [3–7]. One patient showed homozygosity in two mutations [3].

The disease is caused by a decrease in the activity of lysosomal enzyme iduronate-2-sulfatase (I2S, EC 3.1.6.13).

The reason for the decrease in the activity of the I2S enzyme are the mutations in the *IDS* gene that codes this enzyme. The *IDS* gene is localized on the long arm of chromosome X (locus - Xq28); it consists of 9 exons and has a length of 28.3 kb. To date, more than 600 mutations in the *IDS* gene have been described. More than half of the mutations are point mutations, 25% are small deletions, insertions and mutations of the splice site, 20% are occupied by major rearrangements, of which 6–8% are associated with the complete deletion of the *IDS* gene. Located next to the *IDS* gene (90 kb distal), the *IDS2* pseudogene, which has a high degree of homology with the *IDS* gene, makes molecular diagnosis of type II mucopolysaccharidosis difficult.

According to studies, it is difficult to establish unambiguous genotypic-phenotypic correlations in Hunter syndrome, since most mutations are unique for each family, and even patients with the same mutation may have phenotypes of different severity. The only exception is patients with complete deletion of the gene. The clinical symptoms of these patients are characterized by severe manifestations of the disease with early involvement of the nervous system.

Deficiency of iduronate-2-sulfatase leads to the accumulation in different tissues of two types of glycosaminoglycans (GAG), namely heparan sulfate and dermatan sulfate, which causes the multisystem pathology.

The appearance of clinical symptoms in Hunter syndrome is usually noted at the end of the first, beginning of the second year of life. The disease is characterized by a progredient course. Distinguishing signs of the disease from other types of mucopolysaccharidosis, along with the recessive X-linked type of inheritance, are the absence of corneal opacity and the presence of nodular-papular eruptions on the skin in a number of patients, mainly in the area of shoulder blades, the outer and lateral surfaces of shoulders and thighs. These changes are caused by the deposition of lipids and glycosaminoglycans in the dermis. Hunter syndrome is also characterized by low growth, changes in facial features such as “gargoyleism”, skeletal anomalies (multiple dysostosis), cardiovascular pathology (cardiomyopathy, cardiac valve disease, narrowing of the coronary arteries, cardiac rhythm disturbance), obstructive airway disease (obstructive sleep apnea, decreased vital capacity of the lungs), hepatosplenomegaly, joint stiffness, umbilical or inguinal and inguinal-scrotal hernia, retinitis pigmentosa, and progressive conductive or neurosensory deafness [2].

There are mild and severe forms of Hunter syndrome. Patients with the mild form account for approximately one third of all cases. This form is characterized by a later manifestation of clinical symptoms (usually at 2–4 years of age), normal intelligence and longer life expectancy (usually more than 60 years). Patients with the mild form of Hunter syndrome are able to attend regular schools, successfully complete higher education institutions and successfully work in the specialty, occupying even leading positions. They are able to marry and have a healthy offspring. The purpose of this article is to demonstrate a rare case of Hunter syndrome in a girl caused by a mutation in the *IDS* gene inherited from the mother and the presence of the paternal chromosome X with partial deletion in the long arm region.

## Case presentation

Girl M. 4 years old, entered the clinic with complaints of parents for growth retardation, pain in the lower limbs and stiff joints. The genealogy analysis found that the marriage was unrelated, parents were young and healthy, the girl was the only child in the family.

The girl was from the first pregnancy, complicated by an acute respiratory viral infection in the first trimester. The birth was at 40 weeks of pregnancy. Body weight at birth was 3170.0 g, body length was 52 cm. Early motor development proceeded with a slight delay: she began to support the head at the age of 2.5 months, sit at 9 months, and walk at 15 months. The first words began to be pronounced at the age of 12 months.

At the age of 18 months, there were complaints about the short stature of the child, stiffness of the joints. After analyzing the karyotype, which revealed a partial deletion of the long arm of chromosome X - 46, X, del (X) (q 22.1), Turner syndrome was diagnosed. However, due to the presence of a Hurler-like facial phenotype, a genetic doctor suspected type I mucopolysaccharidosis (Hurler syndrome). The study of GAG urine by the method of one-dimensional electrophoresis revealed an increased renal excretion of heparan and dermatan sulfates, which is typical for mucopolysaccharidosis I, II and VII types.

**Fig. 1:**
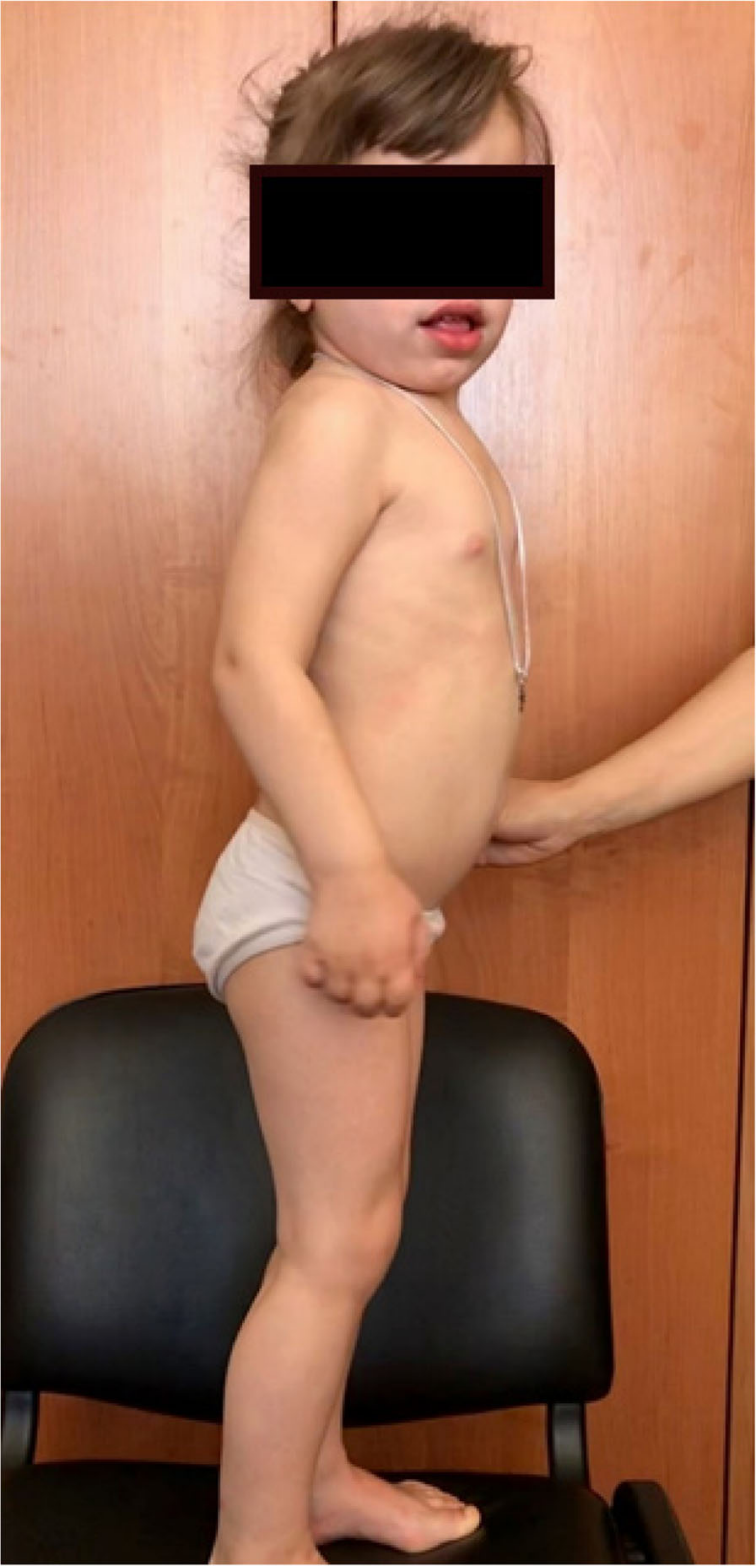
A 4-year old girl with Hunter syndrome

When the girl was admitted to the clinic, her indicators of physical development were disharmonious: the body length (100 cm) corresponded to 3–10 percentile; body weight (17 kg) 90–97 percentile; the head circumference (54 cm) indicated macrocephaly and was above 97 percentile. Pronounced phenotypic features were noted (Fig. 1): rough facial features, sunken nose, full lips, eye hypertelorism, macroglossia, short neck, low position of the auricles, stiffness of large and small joints, equino-varus deformity of the feet, kyphosis of thoracic spine. The hair was dark, eyes - brown, cornea is transparent without visual signs of turbidity. The child’s psychomotor development corresponded to her age: phrasal speech and adequate reaction to the environment. The muscle tone was moderately reduced. Active and passive movements were limited in large and small joints. Tendon reflexes from the hands and feet were alive, pathological reflexes were absent. She was able to walk independently. The examination of the heart revealed a sinus tachycardia with a heart rate of 133–143 per minute, normal position of the electrical axis of the heart, incomplete blockade of the right leg of the bundle, and a slight decrease in the repolarization processes in the myocardium in the form of the T wave flattening. The echocardiogram detected degenerative changes of the mitral and aortic valves with insignificant stenosis, the presence of diagonal trabeculae in the cavity of the left ventricle, and normal dimensions of the heart cavities.

The ultrasound scanning of the abdominal organs and kidneys revealed hepatic splenomegaly (6.5 and 3 cm, respectively) with diffuse changes in the liver parenchyma and a large number of enlarged lymph nodes in the gates of the liver and mesenteric lymph nodes. An increase in the gallbladder and thickening of its wall were also revealed. An increase in kidney volume and bilateral nephroptosis was diagnosed.

The ultrasound examination of the pelvic organs showed the location of the uterus in a typical place, its outline was even and drop-shaped, and the structure of the myometrium was homogeneous. At the site of the projection of the right ovary, the avascular structure was of somewhat reduced echogenicity, an oval shape, 1.0 × 0.5 cm in size with a clear and even contour of the homogeneous structure. The left ovary could not be clearly visualized.

The radiographic examination of the hands revealed coarsening and expansion of the phalanx, hypoplasia of the terminal phalanges, and a backlog of bone age for 1.5 years. Radiography of the shins and knee joints showed valgus deformation of the shins of the legs, flattening of the epiphyses, and osteoporosis.

Radiography of the hip joints, thoracic and lumbar spine noted asymmetry of the pelvic bones with a decrease in the head of the femur; flattening of the thoracic vertebrae and their cuboid shape in the lumbar region. All these x-ray changes, according to the conclusion of a radiologist, are characteristic of mucopolysaccharidosis, mostly of types II and I.

The examination of the oculist revealed a high degree of myopia with astigmatism of both eyes.

Clinical blood and urine tests revealed no pathology. Biochemical indicators, reflecting the state of the main types of metabolism, were normal.

The results of the study of the amino acid spectrum of blood serum and urine corresponded to physiological values. The level of thyroid hormone, thyroid stimulating hormone and somatomedin C in the blood serum was normal.

The study of lysosomal enzymes showed normal alpha-L-iduronidase activity and a sharp decrease in the activity of iduronate sulfatase in dry blood stains: 0.001 μM/l/h, at a rate of 2.5–50 μM/l/h. The result of molecular genetic analysis in exon 9 of the *IDS* gene revealed a new, non-registered (in the international database of HGMD professional) deletion in the hemizygous state. This deletion was not detected in the girl’s father, but was detected in the mother in a heterozygous state.

## Discussion and conclusions

Thus, the anamnestic data (normal weight-growth parameters at birth, small delay in motor development, normal intelligence), a set of phenotypic signs (low body lengths, formed during the growth of the child, to about 1.5 years, a “Hurler-like” phenotype, contractures of large and small joints) and examination results (degenerative changes in mitral and aortic valves with minor stenosis, indices of radiographic methods of research the poorly visualized ovaries, according to ultrasound of the pelvic organs, cytogenetic analysis and the fractional composition of the GAG urine), were most consistent with two diagnoses: the Turner syndrome and mucopolysaccharidosis with the “Hurler-like phenotype.” For the final diagnostics, the exclusion of diseases characterized by similar clinical symptoms was required.

The “Hurler-like” phenotype and the high renal excretion of GAG (mainly heparin- and dermatansulfates) necessitated a differential diagnosis between the three clinical variants of type I mucopolysaccharidosis (Hurler, Hurler-Scheie and Scheie syndromes), as well as Sly and Hunter syndromes (VII and II types of mucopolysaccharidosis). The normal intelligence of the girl allowed excluding the first clinical variant of the Hurler syndrome and the Sly syndrome. The reference activities of lysosomal hydrolase ά-L-iduronidase and β-glucuronidase in dry blood spots served as the basis for finally rejecting all three clinical variants of the Hurler and Sly syndromes.

Extremely low activity of lysosomal enzyme iduronate sulfatase (0.001 μM/L/h, at a rate of 2.5–50 μM/L/h) indicated the diagnosis of Hunter syndrome or Mucopolysaccharidosis type II. The normal intelligence of the girl allowed diagnosing the mild form of the disease.

For the final confirmation of the diagnosis and reasons for the lack of a corrective effect of the other *IDS* gene, a molecular genetic study was required.

Direct DNA sequencing by Sanger carried out a DNA analysis of nine coding exons of the *IDS* gene and adjacent intron regions. In the course of molecular genetic analysis, deletion of c.1436_1440AGCCG in the hemizygous state was observed in exon 9 of the *IDS* gene. The mutation was not previously described in the HGMD professional, however, this deletion results in a shift in the reading frame and formation of a premature stop codon at the c.1491_1493 position of the DNA strand, which corresponds to the 498-protein chain codon. As a result, peptide breakdown occurs earlier at 53 triplets compared to the norm, which indicates the pathogenicity of this mutation.

**Fig. 2:**
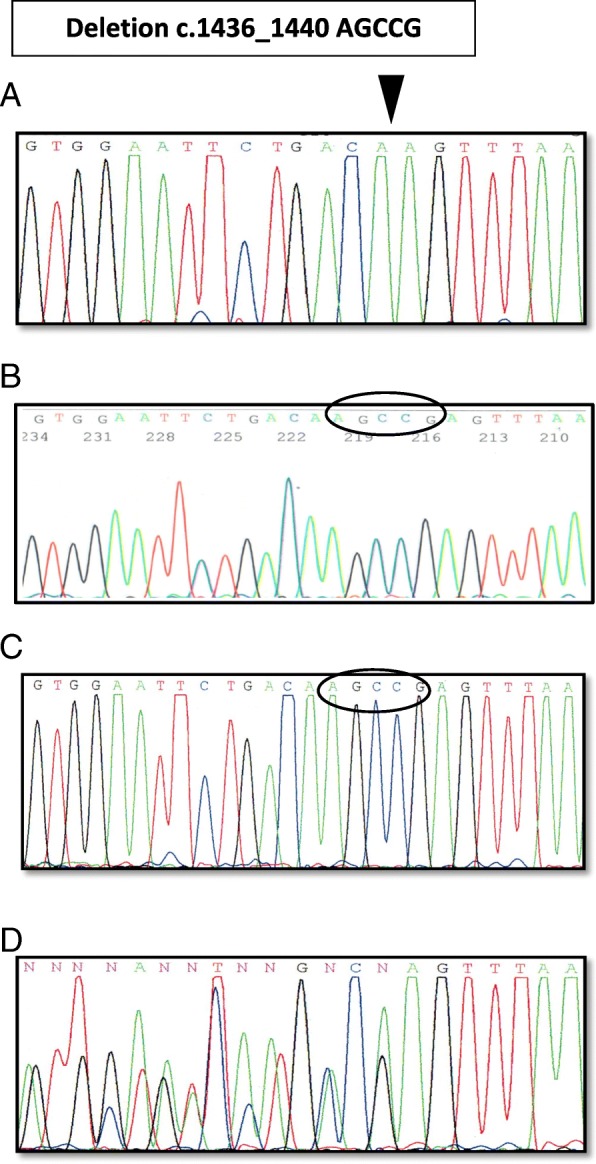
The results of direct sequencing by Sanger. The Fragment of exon 9 of the *IDS* gene: **a**) the proband: deletion p.1436_1440 AGCCG in the “homozygous” state; **b**) normal sequence according to reference NM_000202.5; **c**) the father: a normal variant of the sequence of the *IDS* gene; **d**) the mother: deletion p.1436_1440 AGCCG in the heterozygous state

When performing a family analysis, the deletion of c.1436_1440AGCCG was not detected in the girl’s father, but was detected in the mother in a heterozygous state (Fig. 2).

**Fig. 3:**
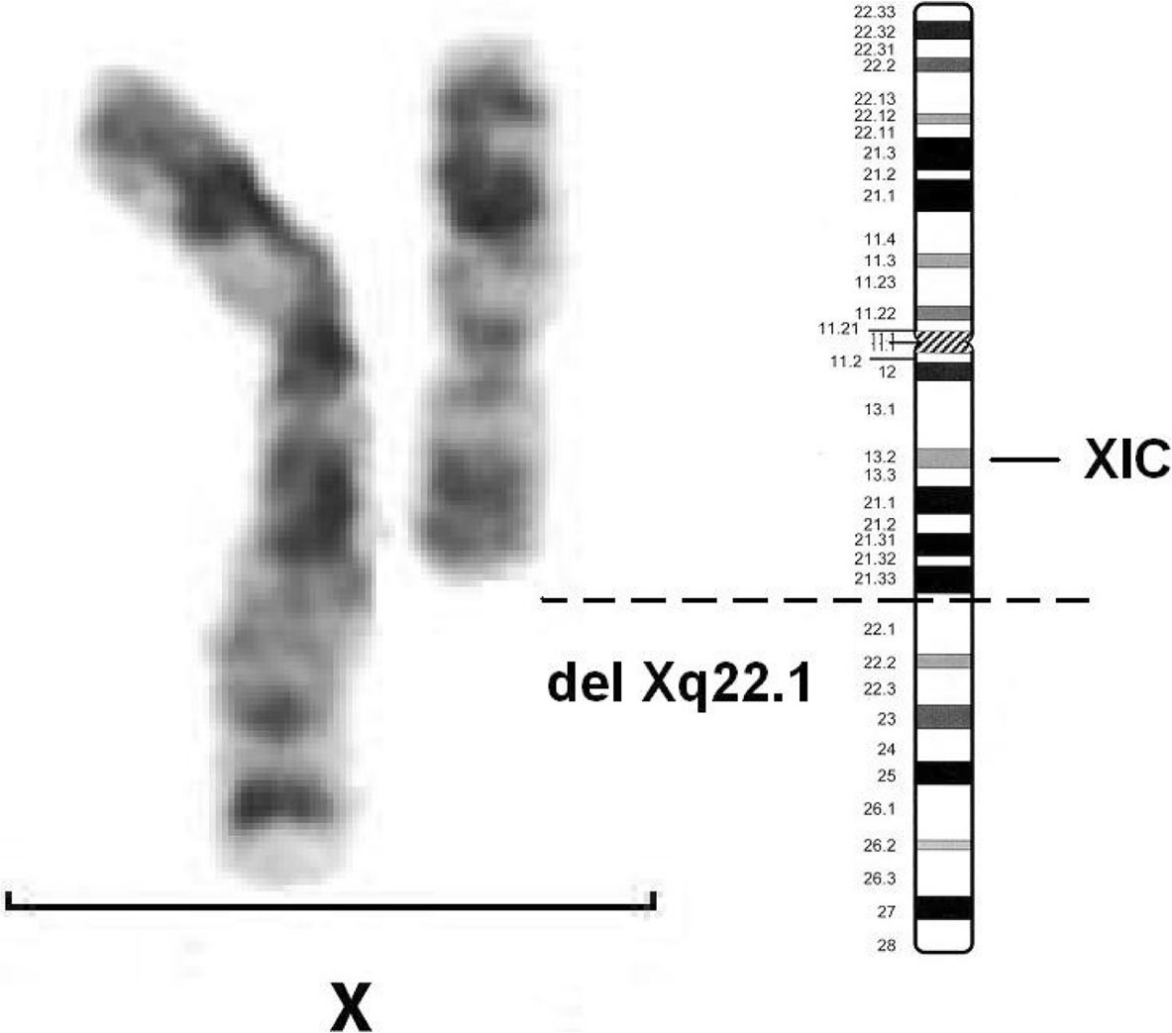
Xq22.1 deletion in the girl (GTG-banding). XIC - X inactivation center, located proximal to the breakpoint; its presence suggests that the rearranged chromosome might be inactivated

During the cytogenetic study, the girl was found to have a deletion of approximately half of the long arm of chromosome X - 46, X, del (X) (q22.1) (Fig. 3). The mother’s chromosomes X had no structural damage, however, mosaic monosomy of chromosome X was revealed: the woman’s karyotype was 45, X [3] / 46, XX [17]. Subsequent FISH study confirmed the presence of mosaic aneuploidy of chromosome X of low level in the mother with the presence of cells 45, X and 47, XXX in 5.2 and 1.3%, respectively, which may indicate the instability of chromosome X carrying a mutation or genome as a whole [8].

**Fig. 4:**
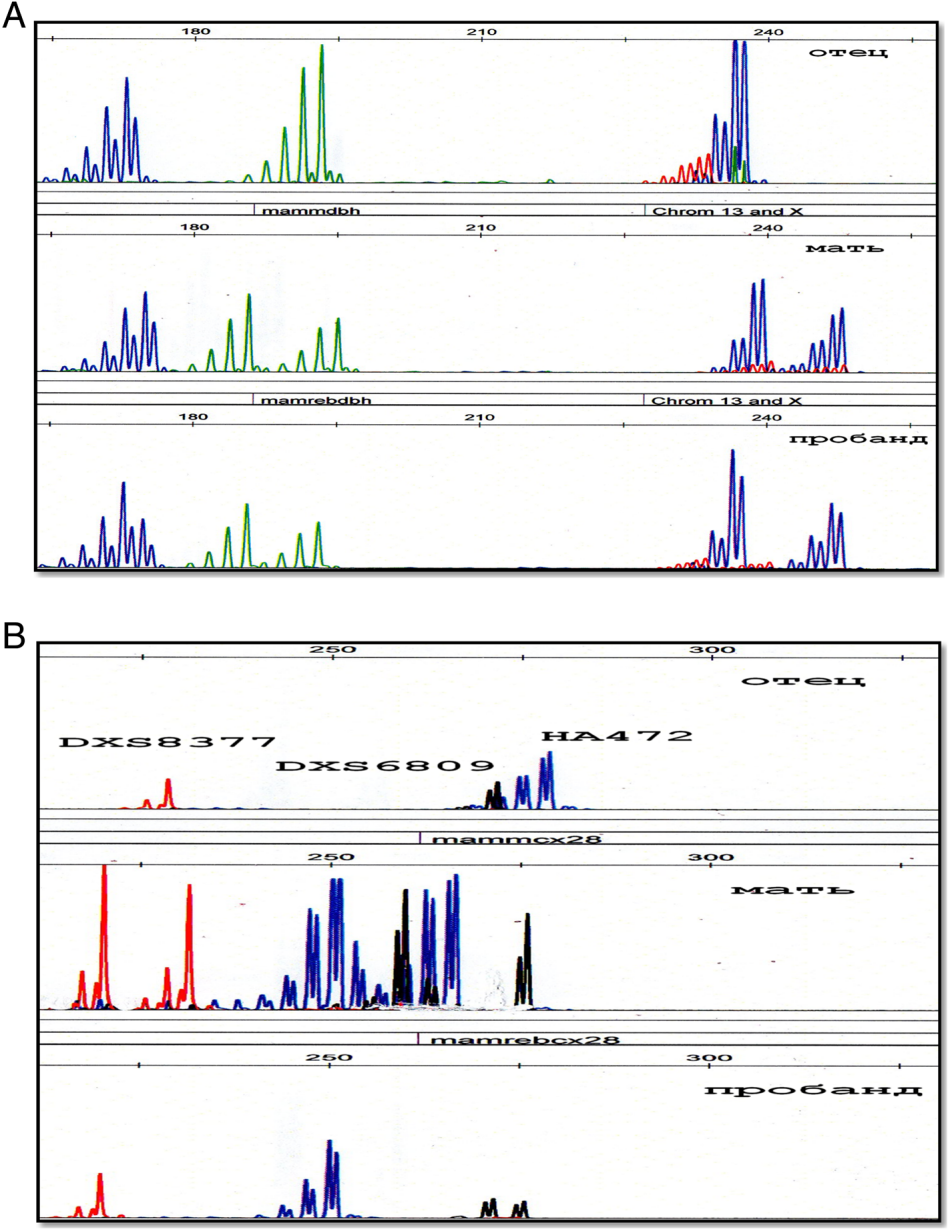
**a**) Family analysis of the STR-loci of the X chromosome located inside the *DMD* gene (localization Xp21.2-p21.1). **b**) Family analysis of STR-loci of the X chromosome: DXS8377, HA472 - localization Xq28; DXS6809 - localization Xq22. The absence of the paternal material of chromosome X is distal to Xq22

**Fig. 5:**
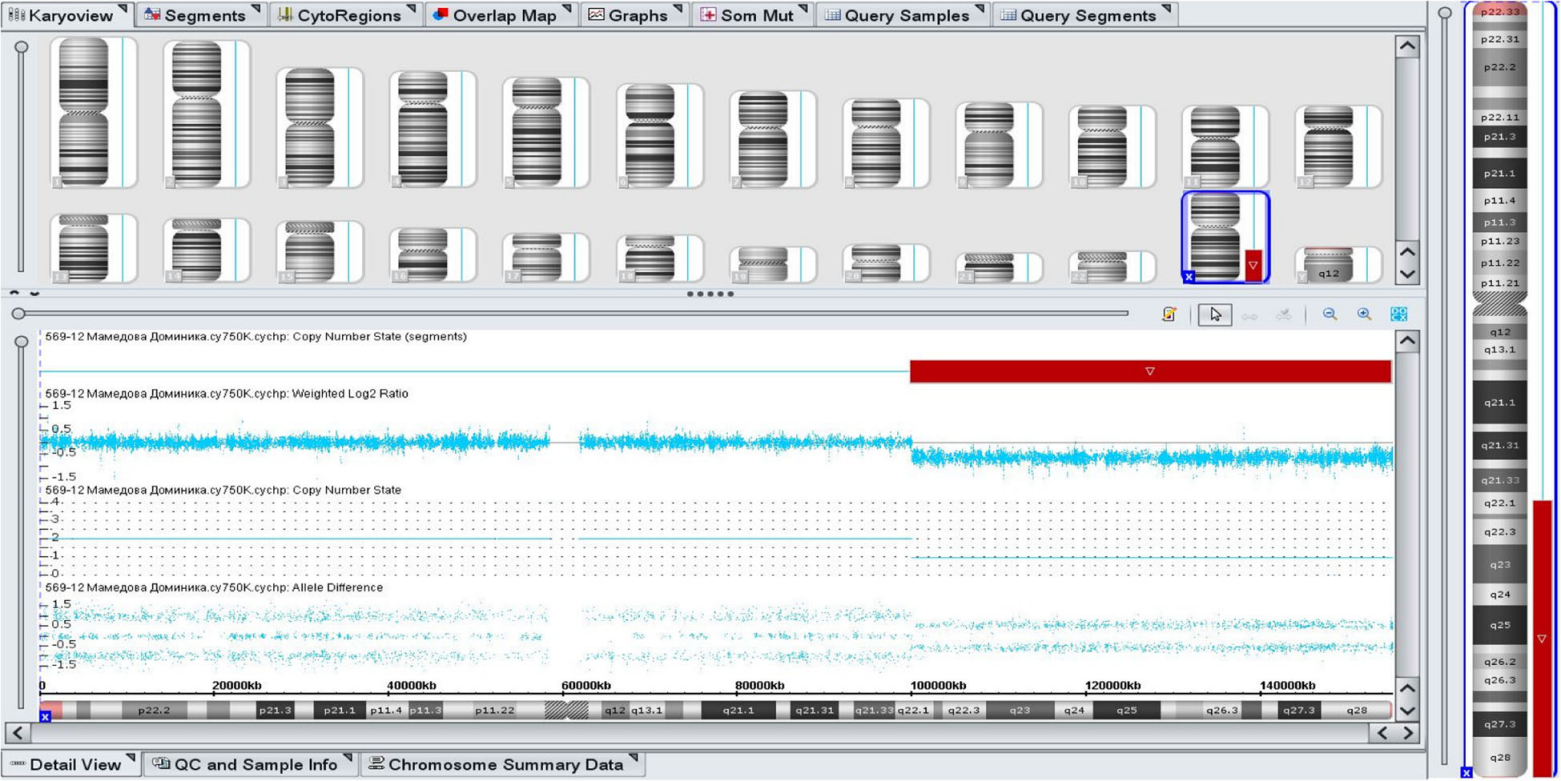
The result of micro-matrix analysis. A deletion of the long arm (q) of chromosome X from position 100,028,096 to position 155,233,098, capturing regions Xq22.1-q28, was detected. Size: 55205002 bp

To determine the points of rupture of the chromosome X deletion and its parent origin, an analysis was made of the STR loci of chromosome X and the microarray analysis. The analysis of STR loci stated that the father’s X chromosome was deleted in the proband and the point of disruption was distal to Xq22 (Figs. 4 and 5). As can be seen from Figs. 4 and 5, the girl has the material of the paternal X chromosome at the loci Xp21 and Xq22, but the material is absent from the Xq28 loci. The microarray analysis revealed a deletion of the long arm of chromosome X from position 100,028,096 to position 155,233,098, the catching regions Xq22.1-q28, the length of 55,205,002 bp. There were 306 OMIM-annotated genes in the deletion area, including the *IDS* gene. A partial deletion of the long arm of the father’s chromosome X, most likely, occurred de novo.

It is known that structurally damaged chromosomes X (having deletions or duplications) in the female body are in the inactivated state, due to selective cellular selection. Thus, in our case, father’s chromosome X with a deletion including Xq28 loci is in an inactivated state with a high degree of probability, while an intact but carrying *IDS* mutation in chromosome X remained active. So, the *IDS* gene with the mutation is expressed from the maternal chromosome X, which does not have structural defects, as a result, the clinical manifestation of the Hunter syndrome appeared in the girl. On the mechanism of disease formation, our case is most similar to the description of Broadhead DM et al. [7].

Thus, the presence of a combination pathology is confirmed in the girl, which is the Turner syndrome with partial deletion of the long arm of the father’s chromosome X, including Xq28 loci (*IDS* gene localization), and the mild form of Hunter syndrome associated with the *IDS* mutation inherited from the mother. Due to the structural defect of chromosome X, there is a hemizygous state with the *IDS* mutation, which led to the formation of the Hunter syndrome clinical phenotype in the girl.

According to the vital indications, the genetic engineering enzyme-substituting preparation Elapraza (idursulfase) was prescribed to the child at a dose of 0.5 mg/kg of body weight intravenously, once a week. The calculated dose of the drug was 2 vials per 1 intravenous injection.

The family conducted an effective medical genetic counseling. At the next pregnancy (at 10–11 weeks), the mother of the child is recommended prenatal diagnosis (chorionic biopsy) with the determination of the *IDS* gene deletion. The presence of the mosaic monosomy of the mother’s chromosome X also necessitates the study of the fetus karyotype.

## Materials and methods

### Electrophoresis of glycosaminoglycans of urine

The isolation of glycosaminoglycans from urine and electrophoresis of glycosaminoglycans of urine were carried out according to the standard procedure described earlier [9].

### Determination of the activity of lysogenic enzymes in dry spots

The activity of lysogenic enzymes in dry blood spots was determined based on the standard procedure.

### PCR and direct sequencing analysis

Oligonucleotide primers to sites of introns flanking the nine encoding exons of the *IDS* gene were synthesized by the commercial firm “Synthol” (Moscow, Russia). The PCR of all the exons of the *IDS* gene was performed as described previously [10]. For all PCR fragments, direct sequencing by Sanger was performed on the automatic sequencer ABI 3130xls with the use of the Taq Dye Deoxy Terminator Cycle Sequencing Kit, according to the manufacturer’s instructions.

### DNA analysis of STR-loci of chromosome X

The sequence of oligonucleotide primers to loci DXS8377, HA472, DXS6809, DBH was selected according to the GeneLoc database. The PCR was carried out based on the standard procedure. The fragment analysis was performed on the genetic analyzer ABI 3130.

The cytogenetic analysis of the girl and her mother was carried out on the metaphase chromosomes of peripheral blood lymphocytes cultured by the standard method using GTG and CBG staining.
